# Supplementary material for: Lateral habenula glutamatergic neurons projecting to the dorsal raphe nucleus promote aggressive arousal in mice
Source: Nat Commun. 2022 Jul 21;13:4039. doi: 10.1038/s41467-022-31728-z (PMC9304121; doi:10.1038/s41467-022-31728-z)
Supplement: Supplementary file 1 — Supplementary Information [file 41467_2022_31728_MOESM1_ESM.pdf]

Supplementary Information

Lateral habenula glutamatergic neurons projecting to the dorsal raphe nucleus promote aggressive arousal in mice.

Supplementary Figure 1: Resident-intruder aggression and social instigation-heightened aggression.

Supplementary Figure 2: Social instigation-heightened aggression and c-Fos in lateral hypothalamus(LH)-DRN projection neurons.

Supplementary Figure 3: Additional behavioral data of chemogenetic inhibition of LHb-DRN projection neurons and instigation-heightened aggression presented in Fig. 3.

Supplementary Figure 4: Additional behavioral data of optogenetic inhibition of LHb-DRN projection neurons and instigation-heightened aggression presented in Fig. 4.

Supplementary Figure 5: Additional behavioral data of optogenetic activation of LHb-DRN projection neurons presented in Fig. 5.

Supplementary Figure 6: Activation of LHb-DRN projection and aggressive behavior in other animal facility.

Supplementary Figure 7: Additional behavioral data of optogenetic activation of LHb-DRN projection neurons during subthreshold social instigation presented in Fig. 5.

Supplementary Figure 8: Additional behavioral data of optogenetic inhibition of DRN 5-HT neurons and instigation-heightened aggression presented in Fig. 6.

Supplementary Figure 9: Anterograde tracing of DRN neurons that receive input from the LHb.

Supplementary Figure 10: Additional behavioral data of optogenetic activation of DRN-VTA projection neurons presented in Fig. 8.

Supplementary Figure 11: Optogenetic stimulation of DRN-VTA projection

in EYFP control animals.

Supplementary Figure 12: Optogenetic activation of DRN-VTA projection neurons increases aggressive behavior.

Supplementary Table 1: Comparison of c-Fos expression in the left and right LHb and LHb-DRN projection neurons.

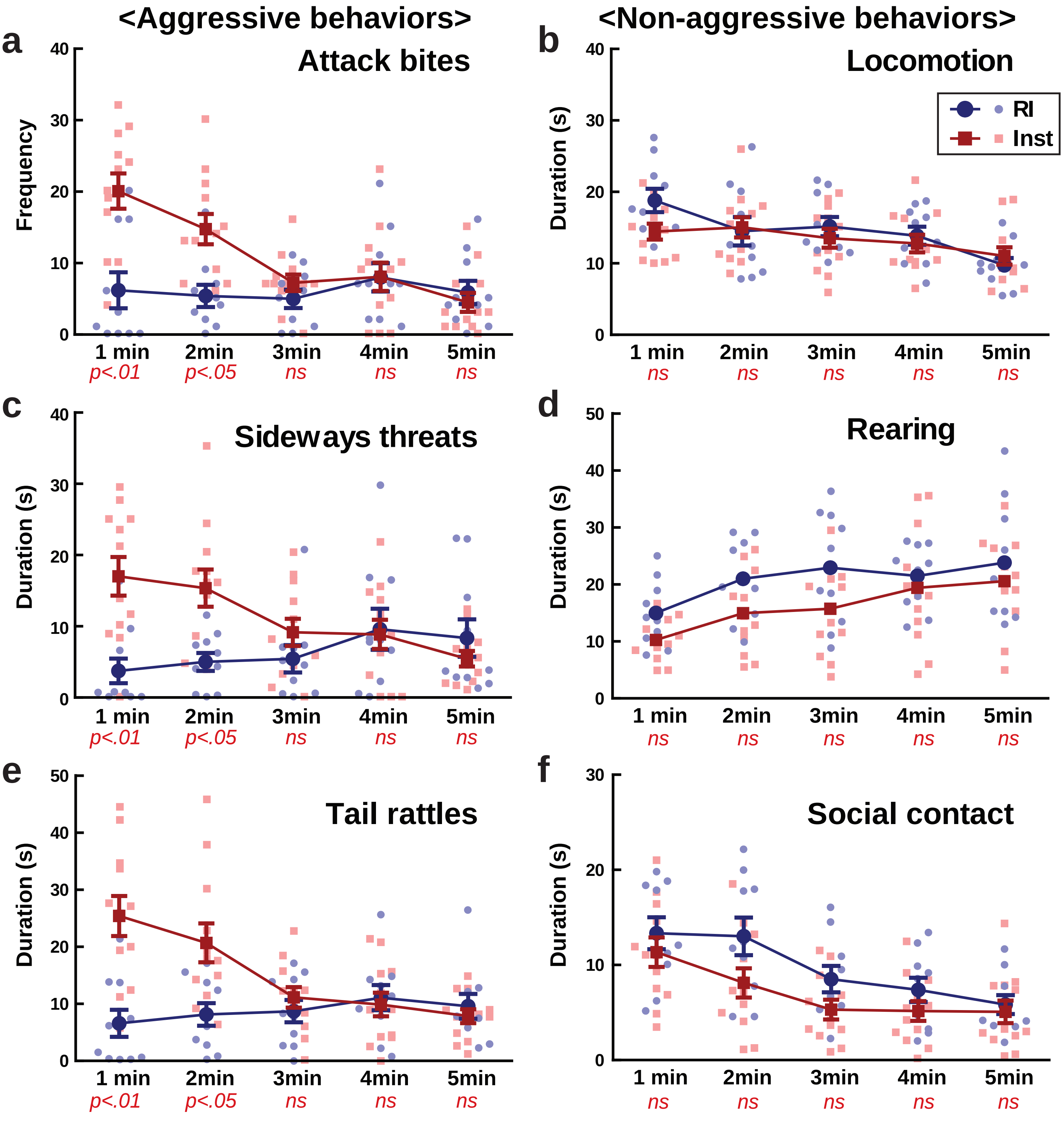

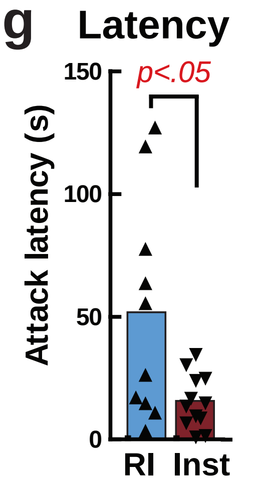

Supplementary Fig. 1 | Resident-intruder aggression and social instigation-heightened aggression. a-f, Detailed behavioral characterization of the effect of social instigation. Standard resident-intruder test group (RI, blue) and social instigation group (Inst, red). Left: Aggressive behaviors including attack bites (**a**), sideways threats (**c**), and tail rattles (**e**). Rght: non-aggressive behaviors including locomotion (**b**), rearing (**d**), and social contact (**f**). Social instigation increased aggressive behaviors in the first 2 min (two-way repeated measures ANOVA with the Geisser-Greenhouse correction, RI n = 10, Inst n = 12, Group × Time interaction, attack bites: F(4,80)=7.009, p<0.0001 (**a**), sideways threats: F(4,80)=6.129, p=0.0002 (**c**), tail rattles: F(4,80)=8.199, p<0.0001 (**e**), posthoc t test with Bonferonni's correction (two-sided)). By contrast, social instigation did not change non-aggressive behaviors (**b,d,f,** two-way repeated measures ANOVA with the Geisser-Greenhouse correction, RI n = 10, Inst n = 12, *n.s.*). **g,** Social instigation significantly reduced attack latency (Mann-Whitney test (two-sided), RI n = 10, Inst n = 12, U=29, p=0.0426). Each bar represents mean value ± SEM. Source data are provided as a Source Data file.

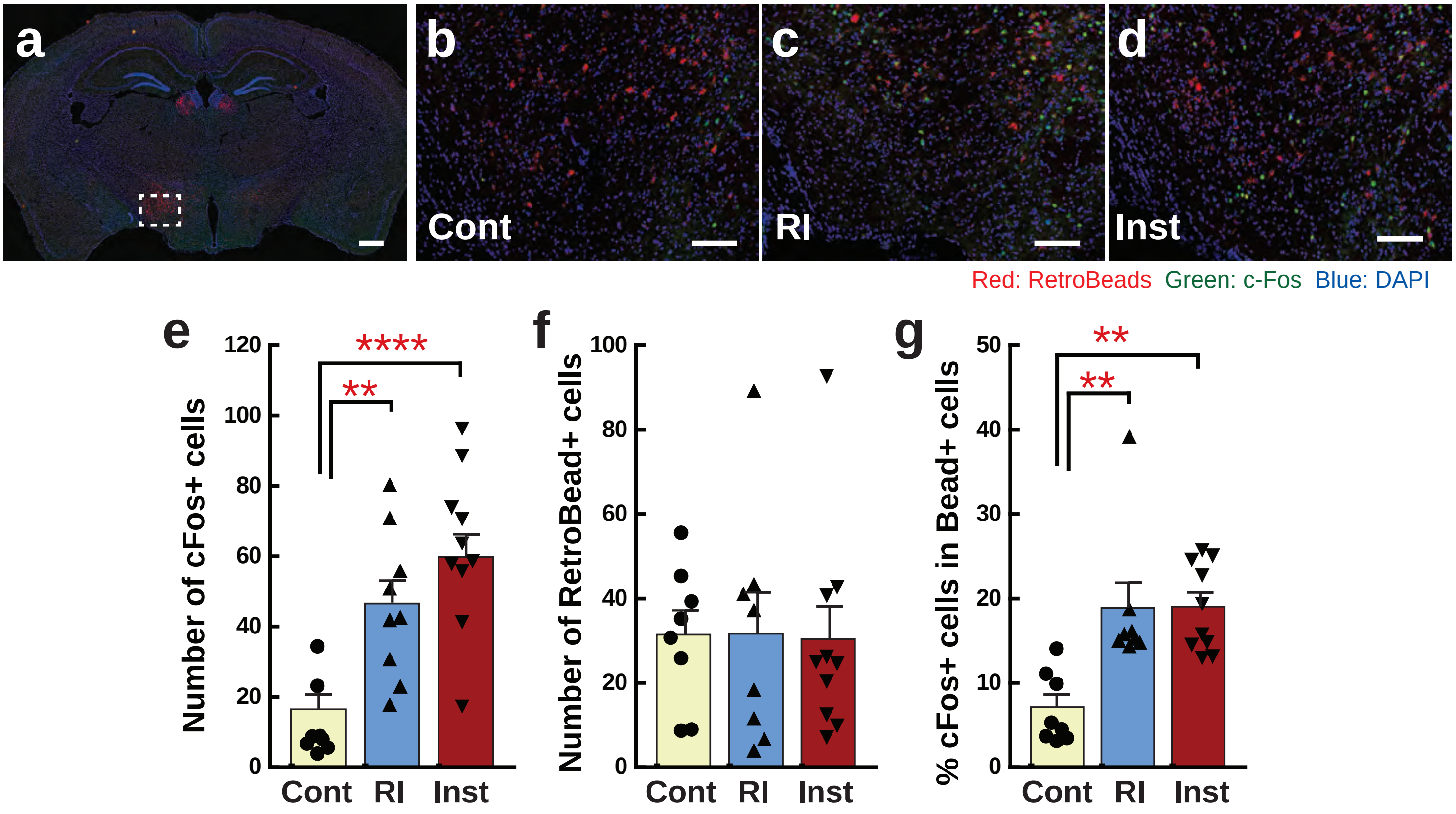

Supplementary Fig. 2 | Social instigation-heightened aggression and c-Fos in lateral hypothalamus(LH)-DRN projection neurons. a, RetroBead-labeled cells (red) were also observed in the LH (scale bar 500 μm). Regular resident-intruder (RI) group was tested for 5 min RI test. Social instigation (Inst) group had 5 min exposure to a caged-instigator male in their homecage prior to 5 min RI test. Control (Cont) animals were kept undisturbed in their homecage. **b-d,** c-Fos expression (green) and RetroBead-labeled cells in the LH of Cont (**b**), RI (**c**), and Inst (**d**) animals. Blue: DAPI (scale bar 100 μm). **e,** Both RI and Inst groups showed higher number of c-Fos expressing cells in the LH compared to Cont (one-way ANOVA, Cont n = 8, RI n = 8, Inst n = 10, F(2,23)=17.35, p<0.0001, posthoc t test with Tukey's multiple comparisons test (two-sided)). **f,** Number of RetroBead-labeled cells in the LHb were not different among groups (one-way ANOVA, Cont n = 8, RI n = 8, Inst n = 10, *n.s.*). **g,** Percent of c-Fos expressing Retrobead-labeled cells were significantly higher in both RI and Inst groups compared to Cont (one-way ANOVA, Cont n = 8, RI n = 8, Inst n = 10, F(2,23)=10.41, p=0.0006, posthoc t test with Tukey's multiple comparisons test (two-sided)) . **p<.01, ****p<.0001. Error bars indicate S.E.M. Source data are provided as a Source Data file.

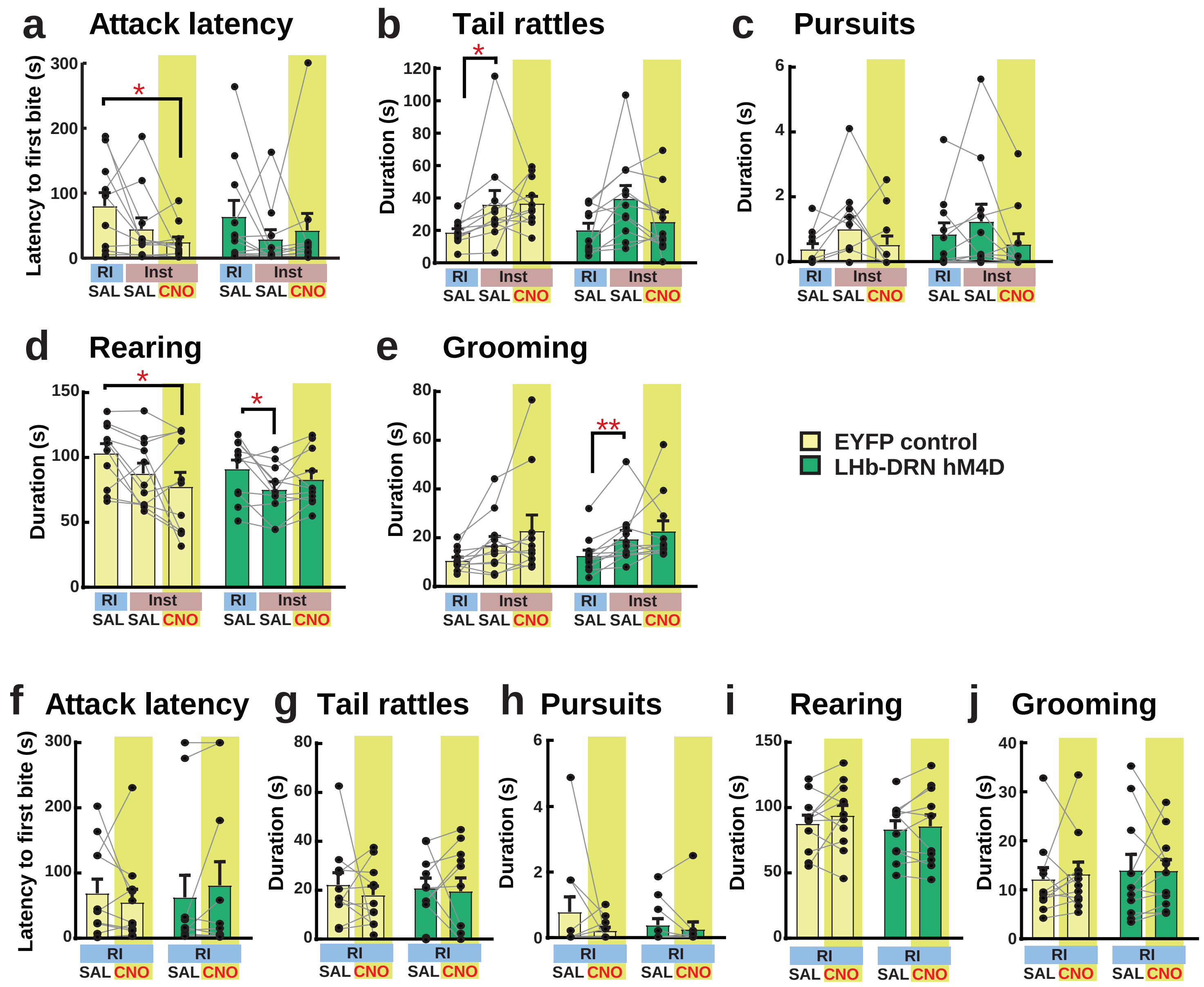

Supplementary Fig. 3 | Additional behavioral data of chemogenetic inhibition of LHb-DRN projection neurons and instigation-heightened aggression presented in Fig. 3.
a-e, Effect of CNO injection on the Inst test. No significant Group × Test-type interaction was observed in attack latency (**a**), tail rattles (**b**), pursuits (**c**), rearing (**d**), and grooming (**e**), but the main effect of Test-type was significant in all behaviors (two-way repeated measures ANOVA with the Geisser-Greenhouse correction, Cont n=11, hM4D n=11, attack latency (**a**): F(1.708,34.15)=4.287, p=0.0269, tail rattles (**b**): F(1.483,29.65)=5.956, p=0.0117, pursuits (**c**): F(1.950,39.01)=3.638, p=0.0366, rearing (**d**): F(1.819,39.39)=7.505, p=0.0024, and grooming (**e**): F(1.364,27.27)=7.926, p=0.0049, posthoc t test with Tukey's multiple comparisons tests (two-sided)). **f-j**, No effect of CNO was observed in the RI test on the attack latency (**f**), the duration of tail rattles (**g**), pursuits (**h**), rearing (**i**), and grooming (**j**) (two-way repeated measures ANOVA, Cont n=11, hM4D n=11, all *n.s.*). Each bar represents mean value ± SEM, and gray line indicates each individual's data. * p<.05, ** p<.01. Source data are provided as a Source Data file.

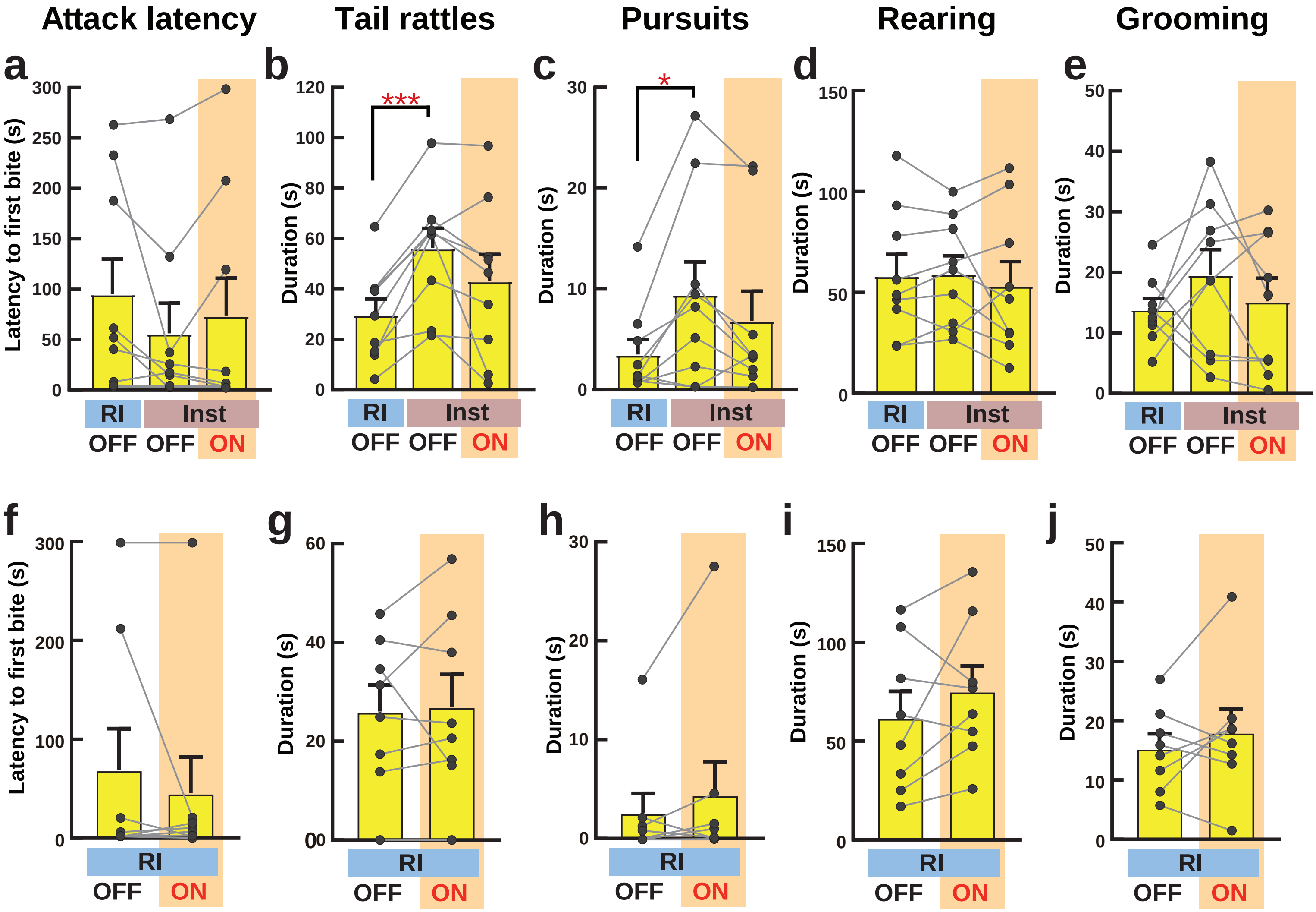

Supplementary Fig. 4 | Additional behavioral data of optogenetic inhibition of LHb-DRN projection neurons and instigation-heightened aggression presented in Fig. 4.

a-e, Effect of light stimulation on the Inst test. There was no significant effect of Inst or light stimulation on the attack latency (**a**, Friedman test (two-sided), n=10, *n.s.*). By contrast, significant increase of aggressive behavior was observed in Inst+OFF but not Inst+ON compared to RI+OFF (tail-rattles (**b**): one-way repeated measures ANOVA, n=9, $F(2,16)=10.95$, $p=0.0026$, posthoc t test with Tukey's multiple comparisons tests (two-sided), pursuits (**c**): Friedman test (two-sided), n=10, Friedman statistic=6.889, $p=0.0307$, posthoc test with Dunn's multiple comparison test (two-sided)). There were no group differences in the duration of rearing (**d**) or grooming (**e**) (one-way repeated measures ANOVA, n=9, both *n.s.*). f-j, No effect of light stimulation was observed in the RI test on the attack latency (**f**), duration of sideways threats (**g**), pursuits (**h**), rearing (**i**), and grooming (**j**) (paired t test (two-sided), n=9, all *n.s.*). Each bar represents mean value ± SEM, and gray line indicates each individual's data. * $p<.05$, *** $p<.001$. Source data are provided as a Source Data file.

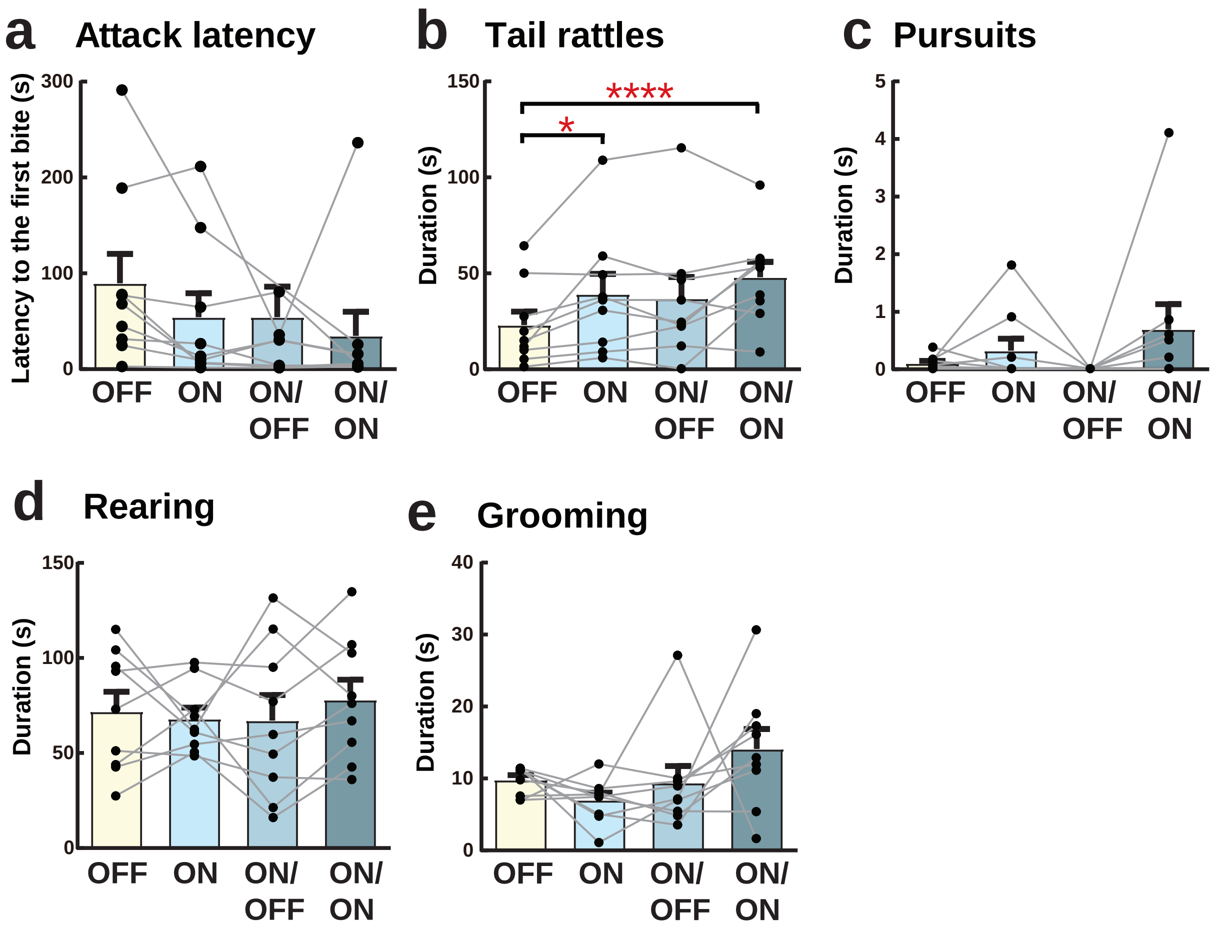

Supplementary Fig. 5 | Additional behavioral data of optogenetic activation of LHb-DRN projection neurons presented in Fig. 5. a, Significant effect of light stimulation was not observed in the attack latency (one-way repeated measures ANOVA, n = 9, *n.s.*). **b-e,** Detailed behavioral analysis data. In addition to attack bites and sideways threats (Fig. 4), tail rattles were significantly increased in ON and ON/ON sessions compared to OFF (**b,** one-way repeated measures ANOVA, n = 9, $F(3,24)=7.497$, $p<0.0001$, post hoc t test with Tukey's multiple comparisons test (two-sided)). No effect of light stimulation was observed in pursuits (**c**), rearing (**d**), and grooming (**e**) (two-sided Friedman test (**c**) or one-way repeated measures ANOVA (**d,e**), n = 9, all *n.s.*). Each bar represents mean value ± SEM, and gray line indicates each individual's data. * $p<.05$, ** $p<.01$. Source data are provided as a Source Data file.

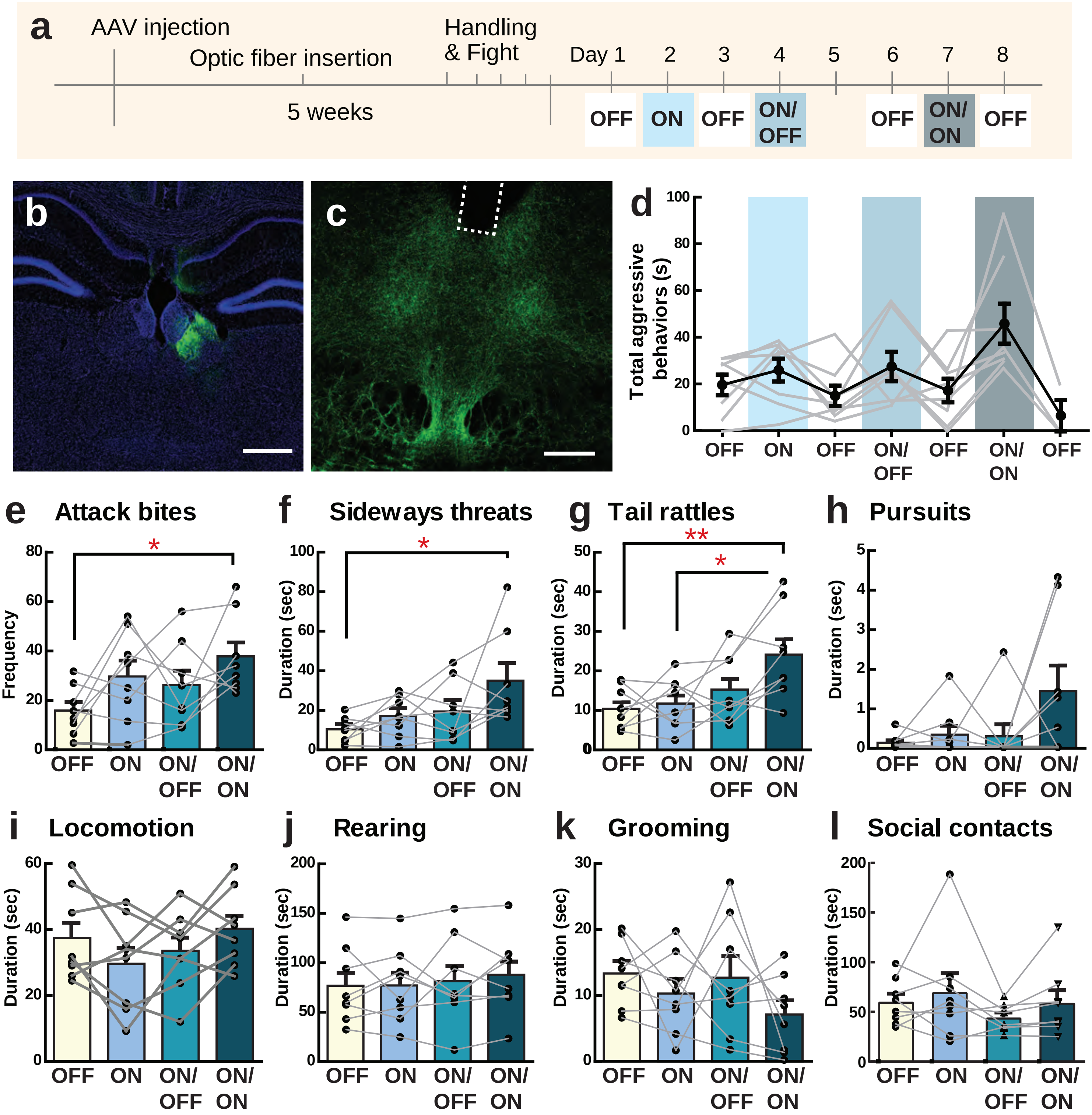

Supplementary Fig. 6 | Activation of LHb-DRN projection and aggressive behavior in other animal facility. **a,** Time line of this experiment. Experiment was conducted in the animal facility of the Icahn School of Medicine at Mount Sinai. **b-c,** AAV2-hSyn-ChR2-EYFP virus was injected into unilateral LHb, and optic fiver was inserted into the DRN. Expression of ChR2-EYFP (green) was observed in the LHb (**b**, scale bar 500 um), and the projection terminals were observed in the DRN (**c**, scale bar 200 um). Animals that express ChR2 in the left and right LHb were combined because there was no difference between the sides. Light stimulation schematics were same as Fig. 4. **d,** Duration of total aggressive behaviors in each session. Black line indicates average data of 9 animals (Mean ± SEM), and gray line indicates each individual's data. **e-l,** Detail behavioral analysis showed that ON/ON session significantly increased aggressive components of behaviors compared to OFF sessions (one-way repeated measures ANOVA, n = 8, attack bites: $F(3,21)=3.330$, $p=0.0392$ (**e**), sideways treats: $F(3,21)=4.141$, $p=0.0188$ (**f**), tail rattles: $F(3,21)=5.993$, $p=0.0041$ (**g**), pursuit: *n.s.* (**h**), post hoc t test with Tukey's multiple comparisons test (two-sided)). **i-l,** There were no effect of light stimulation on non-aggressive behaviors (locomotion (**i**), rearing (**j**), grooming (**k**), and social contacts (**l**) (one-way repeated measures ANOVA, n = 8, all *n.s.*). Each bar represents mean value ± SEM, and gray line indicates each individual's data. * $p<.05$, ** $p<.01$. Source data are provided as a Source Data file.

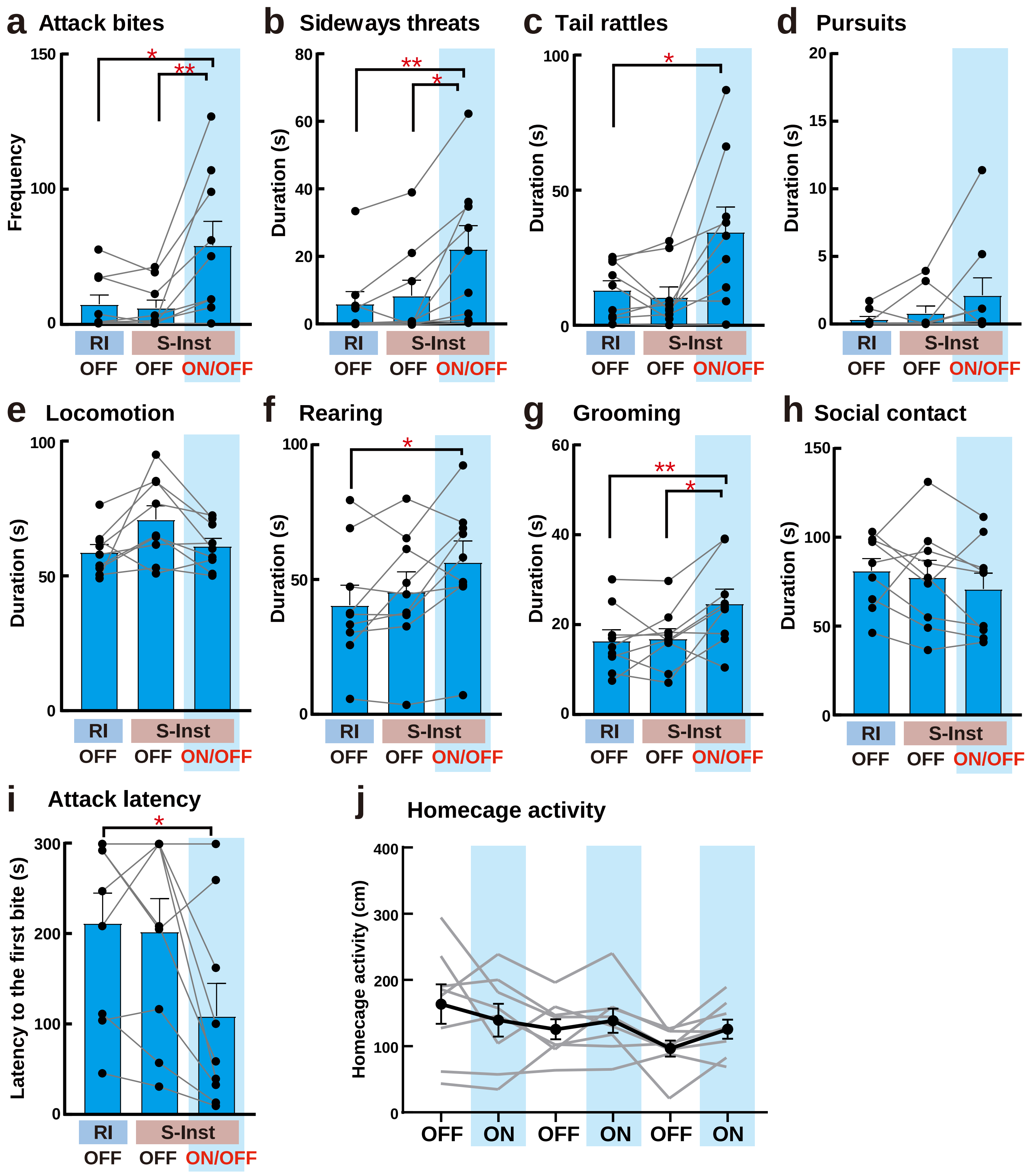

Supplementary Fig. 7 | Additional behavioral data of optogenetic activation of LHb-DRN projection neurons during subthreshold social instigation presented in Fig. 5. Subthreshold social instigation (S-Inst) itself did not affect any behaviors without light stimulation, but a combination of S-Inst with ON/OFF stimulation significantly increased aggressive components of behaviors (**a-d**, Friedman test (two-sided), n = 9, attack bites (**a**): Friedman statistic=12.45, p=0.0005, sideways threats (**b**): Friedman statistic=14.82, p<0.0001, tail rattles (**c**): Friedman statistic=8.222, p=0.0158, but pursuit (**d**): *n.s.*, posthoc test with Dunn's multiple comparison test (two-sided)) as well as some non-aggressive behaviors (**e-h**, one-way repeated measures ANOVA, n = 9, locomotion (**e**): F(1.533,12.26)=4.609, p=0.0398, rearing (**f**): F(1.889,15.11)=5.918, p=0.0136, grooming (**g**): F(1.710,13.68)=8.101, p=0.0062, social contact (**h**): *n.s.*, post hoc t test with Tukey's multiple comparisons test (two-sided)). **i**, There was a significant group difference in the attack latency (one-way repeated measures ANOVA, n=9, F(1.543,12.34)=7.824, p=0.0092, post hoc t test with Tukey's multiple comparisons test (two-sided)). **j**, Spontaneous activity in the homecage was not affected by LHb-DRN activation (paired t test between OFF average and ON average (two-sided), n=8, *n.s.*). Each bar represents mean value ± SEM, and gray line indicates each individual's data. * p<.05, ** p<.01. Source data are provided as a Source Data file.

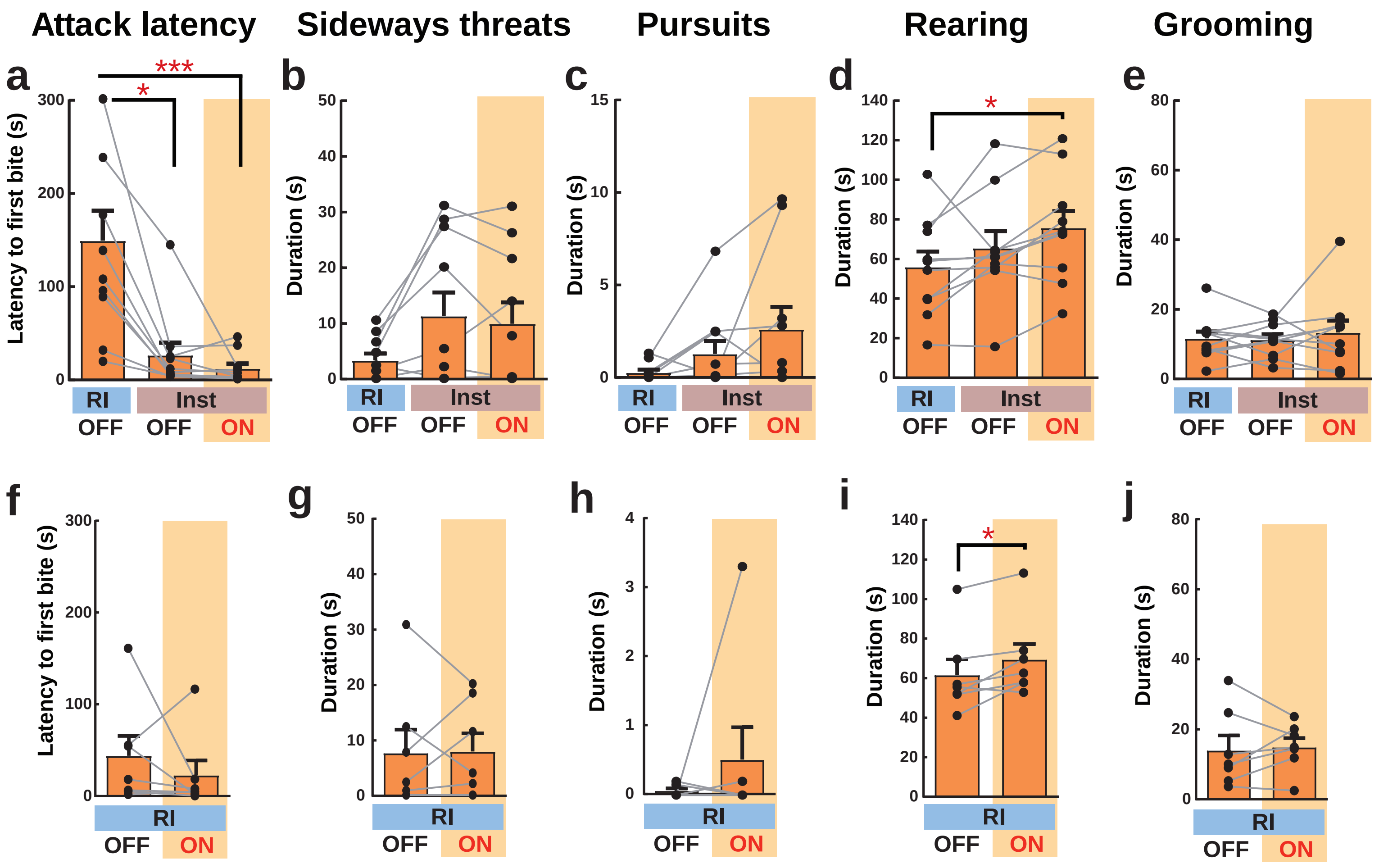

Supplementary Fig. 8 | Additional behavioral data of optogenetic inhibition of DRN 5-HT neurons and instigation-heightened aggression presented in Fig. 6. a-e, Effect of light stimulation on Inst test. Inst reduced attack latency regardless of whether light stimulation was delivered or not (**a**, Friedman test (two-sided), n=10, Friedman statistic=15.80, p<0.0001, posthoc test with Dunn's multiple comparison test (two-sided)). Similarly, there was no effect of light stimulation on other aggressive behaviors (Friedman test (two-sided), n=10, sideways threats: *n.s.* (**b**), pursuits: Friedman statistic=7.630, p=0.0171 (**c**) but no significant group differences by posthoc test with Dunn's multiple comparison test (two-sided)). There was a significant increase of rearing in Inst+ON compared to RI+OFF (**d**, one-way repeated measures ANOVA, n=10, F(1.342,12.08)=6.375, p=0.0201, post hoc t test with Tukey's multiple comparisons test (two-sided)). By contrast no effect of Inst or light stimulation was observed in grooming (**e**, one-way repeated measures ANOVA, n=10, *n.s.*). **j-n**, No effect of light stimulation was observed in the RI test on the attack latency (**f**), duration of sideways threats (**g**), pursuits (**h**), and grooming (**j**) (Wilcoxon matched-pairs signed rank test (**f**,**h**) or paired t test (**g**), both two-sided, n=10, all *n.s.*), except the rearing (**i**, Wilcoxon matched-pairs signed rank test (two-sided), W=26, p=0.0313). Each bar represents mean value ± SEM, and gray line indicates each individual's data. * p<.05, ***p<.001. Source data are provided as a Source Data file.

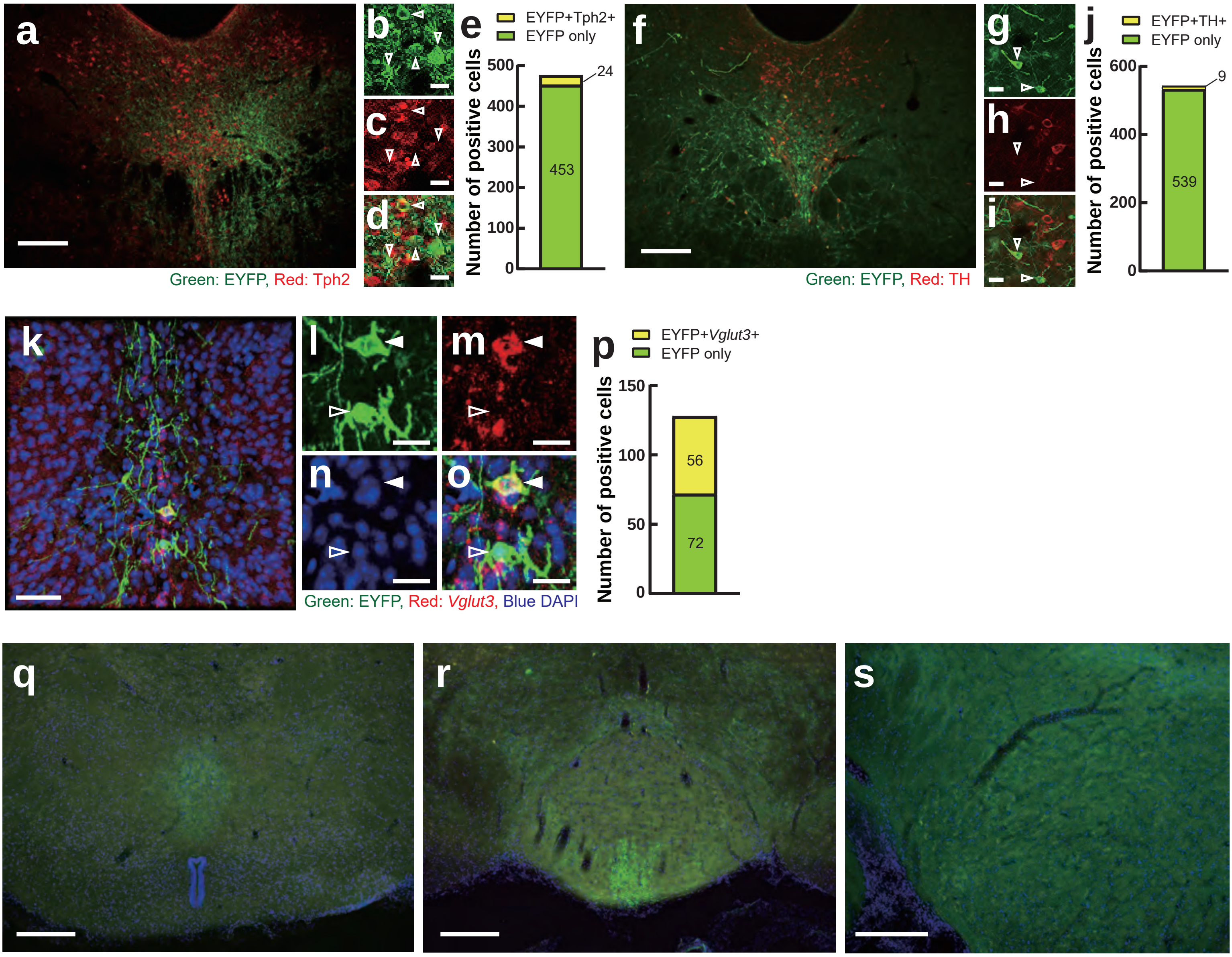

Supplementary Fig. 9 | Anterograde tracing of DRN neurons that receive input from the LHb. AAV1-Cre was injected into unilateral LHb and Cre-dependent AAV2-EF1α-DIO-eNpHR3.0-EYFP (referred below as EYFP) was injected into the DRN (Fig. 6h). **a,** EYFP-expressing cells (green) and Tph2-ir+ cells (red) were observed in the DRN (scale bar 200 μm). **b-d,** Enlarged pictures of EYFP (**b**), Tph2 (**c**), and their marge (**d**) (scale bar 20 μm). Most of EYFP expressing cells (arrow heads) did not colocalize with Tph2+ cells. **e,** Ratio of EYFP+ cells that co-localized with Tph2 (yellow, 24 cells (5.3%)) and without Tph2 (green, 453 cells (94.7%)) in the DRN (n=3 animals). **f,** EYFP-expressing cells (green) and TH-ir+ cells (red) were observed in the DRN (scale bar 200 μm). **g-i,** Enlarged pictures of EYFP (**g**), TH (**h**), and their marge (**i**) (scale bar 20 μm). Most of EYFP expressing cells (arrow heads) did not colocalize with TH+ cells. **j,** Ratio of EYFP+ cells that co-localized with TH (yellow, 9 cells (1.7%)) and without TH (green, 534 cells (98.3%)) in the DRN (n=3 animals). **k,** EYFP-expressing cells (green) and *Vglut3* mRNA positive cells (red) were visualized in the DRN by in situ hybridization (scale bar 50 μm). **l-o,** Enlarged pictures of EYFP (**l**), *Vglut3* (**m**), DAPI (**n**) and their marge (**o**) (scale bar 20 μm). Arrowheads indicate *Vglut3*+ EYFP+ cells (filled) and *Vglut3*- EYFP+ cells (without fill). **p,** Ratio of EYFP+ cells that co-localized with *Vglut3* (yellow, 56 cells (43.8%)) and without *Vglut3* (green, 72 cells (56.2 %)) in the DRN (n=3 animals). **q-s,** EYFP projection terminals. In addition to the VTA and LHb (Fig. 6), EYFP-expressing fibers (green) were observed in the medial mammillary nucleus (**q**, scale bar 200μm), caudal and lateral interpeduncular nucleus (**r**, 200μm), and sparsely in the lateral hypothalamus (**s**, scale bar 200μm). Source data are provided as a Source Data file.

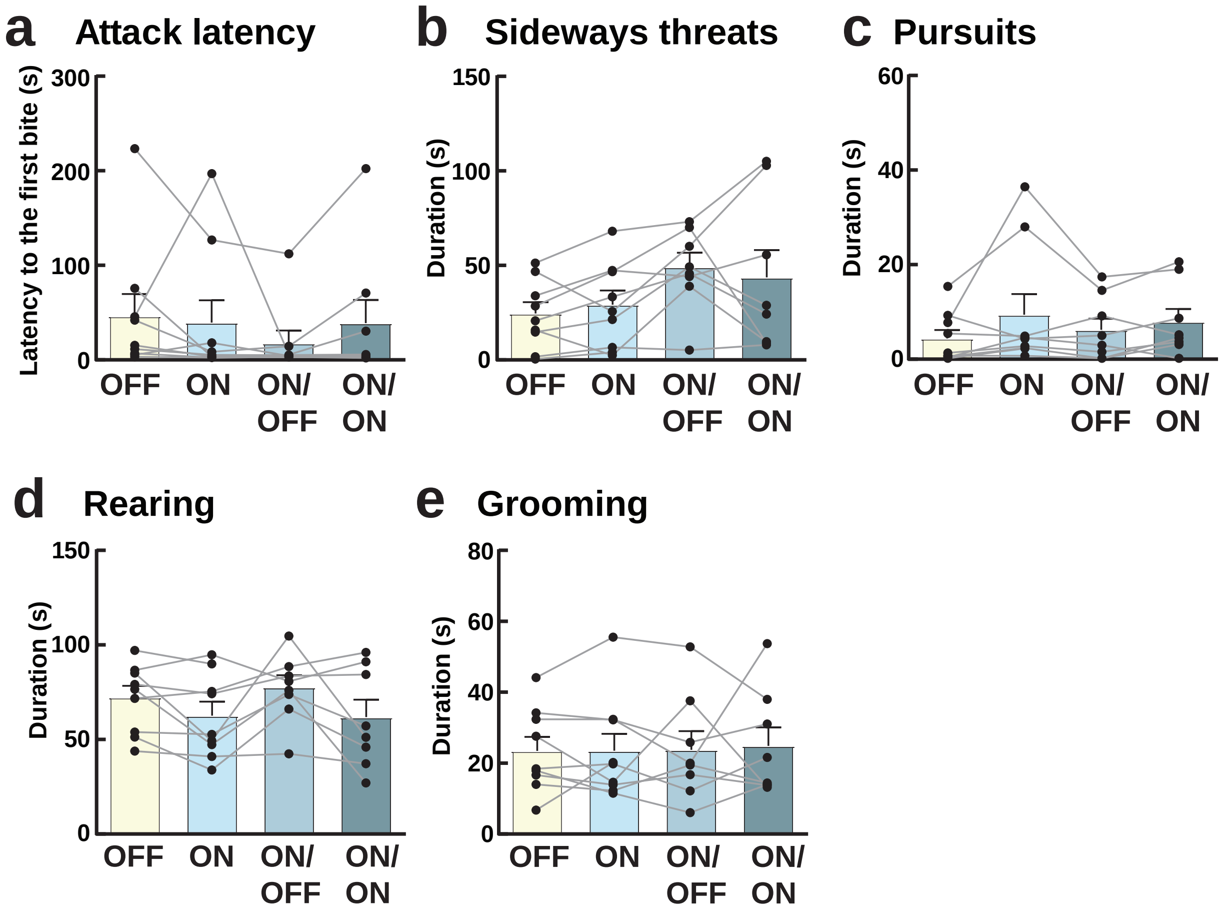

Supplementary Fig. 10 | Additional behavioral data of optogenetic activation of DRN-VTA projection neurons presented in Fig. 8. a, No significant effect of light stimulation was not observed in the attack latency (Mixed-effects analysis (two-sided), n=9, *n.s.*). **b-e,** Detailed behavioral analysis data. No significant effect of light stimulation was observed in these behaviors (Mixed-effects analysis (two-sided), n=9, all *n.s.*). Each bar represents mean value ± SEM, and gray line indicates each individual's data. Source data are provided as a Source Data file.

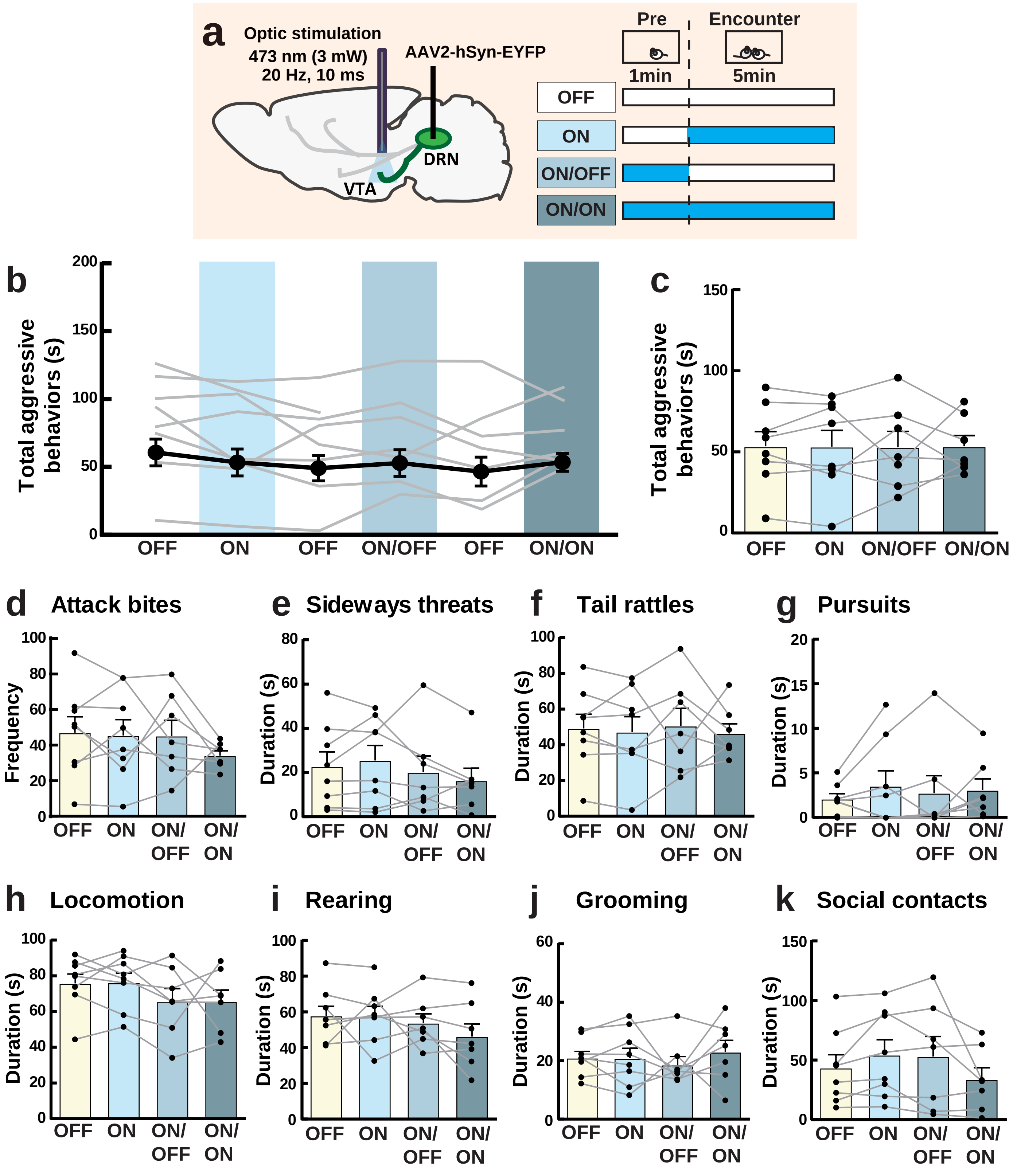

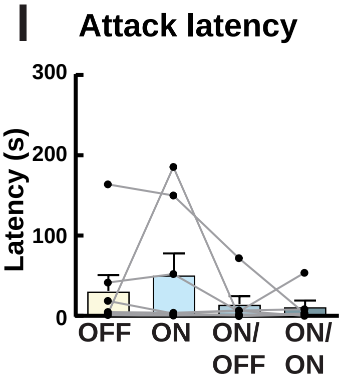

Supplementary Fig. 11 | Optogenetic stimulation of DRN-VTA projection in EYFP control animals. a, Schematics of this experiment. AAV2-hSyn-EYFP was injected in the DRN, and optic fiber was inserted into the VTA. **b-c,** No effect of light stimulation on the duration of total aggressive behavior (Mixed-effects analysis (two-sided), n=8, *n.s.*). **d-k,** Detailed behavioral analysis data also showed no significant effect of light stimulation on neither aggressive and non-aggressive behaviors (Friedman test (**d,l**) or Mixed-effects analysis (**e-k**), both two-sided, n=8, all *n.s.*). **l,** No effect of light stimulation on the attack latency (Friedman test (two-sided), n=8, all *n.s.*).. Each bar represents mean value ± SEM, and gray line indicates each individual's data. Source data are provided as a Source Data file.

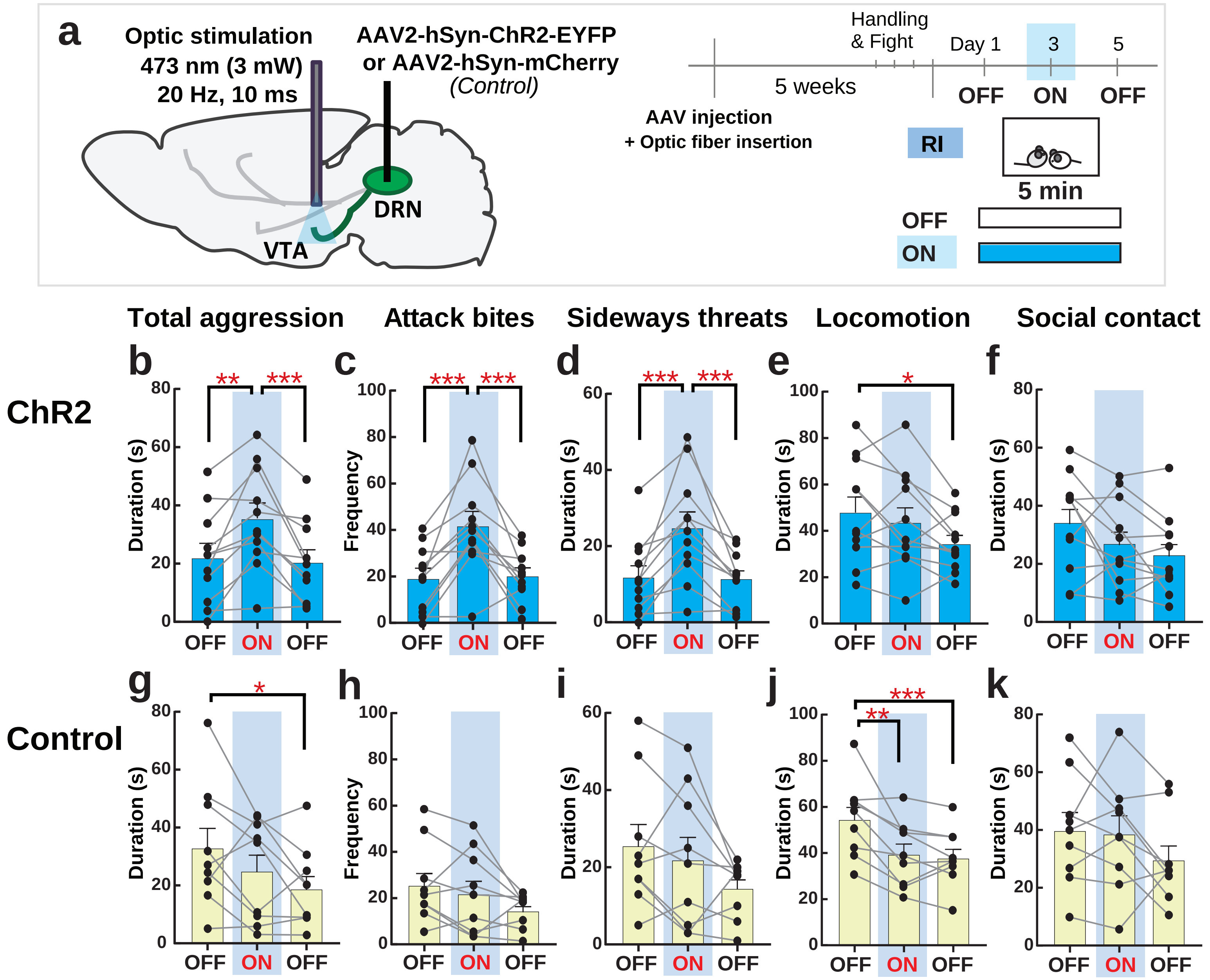

Supplementary Fig. 12 | Optogenetic activation of DRN-VTA projection neurons increases aggressive behavior. a, Schematics and timeline of this experiment. Either AAV2-hSyn-ChR2-EYFP or AAV2-hSyn-mCherry (control) was injected into the DRN. RI test was conducted every other day, optic stimulation was delivered to the DRN on Day 3 (473 nm, 20 Hz, 10 ms pulse, 3 mW). **b-k,** Behaviors of DRN-VTA ChR2 group (**b-f**) and mCherry control group (**g-k**). ON session showed a significant increase in aggressive behaviors compared to OFF sessions in the ChR2 group (**b-d**), but not in mCherry control group (**g-i**) (two-way repeated measures ANOVA, ChR2 n=11, mCherry n=9, Group × Day interaction; total aggressive behaviors: $F(2,36)=5.625$, $p=0.0075$ (**b,g**), attack bites: $F(2,36)=8.281$, $p=0.0011$ (**c,h**), sideways threats: $F(2,36)=4.742$, $p=0.0149$ (**d,i**), posthoc t test with Tukey's multiple comparison tests (two-sided)). By contrast non-aggressive behaviors only showed day-by-day reduction in ChR2 group and mCherry group (two-way repeated measures ANOVA, ChR2 n=11, mCherry n=9, main effect of Day; locomotion: $F(2,36)=14.22$, $p<0.0001$ (**e,j**), social contact: $F(2,36)=4.643$, $p=0.0161$ (**f,k**), posthoc t test with Tukey's multiple comparison tests (two-sided)). Each bar represents mean value ± SEM, and gray line indicates each individual's data. * p<.05, ** p<.01, ***p<.001. Source data are provided as a Source Data file.

Supplementary Table 1 Comparison of c-Fos expression in the left and right LHb and LHb-DRN projection neurons

	# of cFos+ cells		# of Retrobead+ cells		# of c-Fos+ & Retrobead+ cells	
	Left	Right	Left	Right	Left	Right
Control	21.1±4.4	21.3±3.4	2.9±0.9	2.9±0.8	20.2±6.3	24.9±8.4
RI	48.6±5.3	47.4±4.8	11.7±4.5	10.8±3.8	21.9±7.1	23.6±7.0
Inst	49.8±2.4	48.7±2.9	13.4±3.2	14.4±3.7	22.3±8.5	21.9±7.7
