# Supplementary material for: Lateral habenula glutamatergic neurons projecting to the dorsal raphe nucleus promote aggressive arousal in mice
Source: Nat Commun. 2022 Jul 21;13:4039. doi: 10.1038/s41467-022-31728-z (PMC9304121; doi:10.1038/s41467-022-31728-z)
Supplement: Supplementary file 3 — Reporting Summary [file 41467_2022_31728_MOESM3_ESM.pdf]

nature research

Corresponding author(s): Aki Takahashi

Last updated by author(s): May 12, 2022

Reporting Summary

Nature Research wishes to improve the reproducibility of the work that we publish. This form provides structure for consistency and transparency in reporting. For further information on Nature Research policies, see our Editorial Policies and the Editorial Policy Checklist.

Statistics

For all statistical analyses, confirm that the following items are present in the figure legend, table legend, main text, or Methods section.

n/a	Confirmed	
☐	☒	The exact sample size (*n*) for each experimental group/condition, given as a discrete number and unit of measurement
☐	☒	A statement on whether measurements were taken from distinct samples or whether the same sample was measured repeatedly
☐	☒	The statistical test(s) used AND whether they are one- or two-sided<br>*Only common tests should be described solely by name; describe more complex techniques in the Methods section.*
☒	☐	A description of all covariates tested
☐	☒	A description of any assumptions or corrections, such as tests of normality and adjustment for multiple comparisons
☐	☒	A full description of the statistical parameters including central tendency (e.g. means) or other basic estimates (e.g. regression coefficient) AND variation (e.g. standard deviation) or associated estimates of uncertainty (e.g. confidence intervals)
☐	☒	For null hypothesis testing, the test statistic (e.g. *F*, *t*, *r*) with confidence intervals, effect sizes, degrees of freedom and *P* value noted<br>*Give P values as exact values whenever suitable.*
☒	☐	For Bayesian analysis, information on the choice of priors and Markov chain Monte Carlo settings
☒	☐	For hierarchical and complex designs, identification of the appropriate level for tests and full reporting of outcomes
☒	☐	Estimates of effect sizes (e.g. Cohen's *d*, Pearson's *r*), indicating how they were calculated

Our web collection on statistics for biologists contains articles on many of the points above.

Software and code

Policy information about availability of computer code

Data collection	ex vivo electrophysiology recording: pClamp (version 11, Molecular Devices) and Clampex (version 10.3, Molecular Devices).
Data analysis	Behavioral analysis: tanaMove v0.01, a free software established by A. Tanave (http://www.mgrl-lab.jp/tanaMove.html).<br>Microscopic analysis: Imaris (Ver. 9.2.1, Bitplane Inc.), and Photoshop CC 2018 (Ver. 19.0, Adobe Inc.)<br>ex vivo electrophysiology analysis: pClamp (version 11, Molecular Devices), Clampfit (ver. 10.3, Molecular Devices) and Igor Pro (WaveMetrics)<br>Statistic analysis: GraphPad Prism (Ver. 9.3.1, GraphPad Software Inc.)

For manuscripts utilizing custom algorithms or software that are central to the research but not yet described in published literature, software must be made available to editors and reviewers. We strongly encourage code deposition in a community repository (e.g. GitHub). See the Nature Research guidelines for submitting code & software for further information.

Data

Policy information about availability of data

All manuscripts must include a data availability statement. This statement should provide the following information, where applicable:

- Accession codes, unique identifiers, or web links for publicly available datasets
- A list of figures that have associated raw data
- A description of any restrictions on data availability

All data generated and analyzed during this study are available from the supplementary materials.

Field-specific reporting

Please select the one below that is the best fit for your research. If you are not sure, read the appropriate sections before making your selection.

[x] Life sciences [ ] Behavioural & social sciences [ ] Ecological, evolutionary & environmental sciences

For a reference copy of the document with all sections, see nature.com/documents/nr-reporting-summary-flat.pdf

Life sciences study design

All studies must disclose on these points even when the disclosure is negative.

Sample size: Sample sizes were determined based on previous publications (Takahashi et al 2010, 2015, Golden et al 2016, Flanigan et al 2020).

Data exclusions: One male which did not show any aggressive behavior throughout the experiment, 12 animals without ChR2 expression or miss-placement of optic fiber, 1 animal without M4 expression, and 9 animals that did not show instigation-heightened aggression with saline injection in DREADD experiment were excluded from the analysis. No data was excluded for other reasons.

Replication: We replicated the effect of the LHb-DRN optogenetic stimulation in two separate groups of mice independently in different animal facilities (one test in each facility). We also replicated the effect of the and DRN-VTA optogenetic stimulation in two separate groups of mice independently in different animal facilities (one test in each facility). When possible, data was collected using biological replicates (e.g. multiple brain slices per animal analyzed for immunohistochemistry and in-situ hybridization). All attempts at replication were successful.

Randomization: Animals were assigned randomly to control and experimental groups.

Blinding: Although experimenters were not blinded to group allocation for data collection, subsequent offline analysis of behavioral videos was performed blinded to experimental conditions. The experimenter was blinded to experimental conditions for analysis of immunohistochemistry and in situ hybridization.

Reporting for specific materials, systems and methods

We require information from authors about some types of materials, experimental systems and methods used in many studies. Here, indicate whether each material, system or method listed is relevant to your study. If you are not sure if a list item applies to your research, read the appropriate section before selecting a response.

Materials & experimental systems

n/a	Involved in the study	
[ ]	[x]	Antibodies
[x]	[ ]	Eukaryotic cell lines
[x]	[ ]	Palaeontology and archaeology
[ ]	[x]	Animals and other organisms
[x]	[ ]	Human research participants
[x]	[ ]	Clinical data
[x]	[ ]	Dual use research of concern

Methods

n/a	Involved in the study	
[x]	[ ]	ChIP-seq
[x]	[ ]	Flow cytometry
[x]	[ ]	MRI-based neuroimaging

Antibodies

Antibodies used: Rabbit anti-c-Fos (Abcam, polyclonal, ab190289, lot:GR304825-3,1:2000), Goat anti-GFP (Abcam, polyclonal, ab6673, lot:GR3371856-3, 1:3000 or 1:6000), Rabbit anti-GFP (Abcam, polyclonal, ab290, lot:514983, 1:200; Molecular Probes, polyclonal, A11122, lot:1232939, 1:500), Chicken anti-mCherry (Abcam, polyclonal, ab205402, lot:GR3271744-12, 1:2000), Goat anti-Tph2 (Abcam, polyclonal, ab121013, lot:GR3271744-12, 1:1000), Mouse anti-TH(F-11) (Santa Cruz Biotechnology, monoclonal, sc-25269, lot:GR176206-49, 1:1000), Sheep anti-Dig (Roche Diagnostics, polyclonal, 11207733910, lot: 35698000, 1:100), anti-rabbit Alexa488/ anti-rabbit Alexa594/anti-goat Alexa488/anti-goat Alexa680 /anti-chicken Alexa594 (Jackson ImmunoResearch, 1:1250), anti-rabbit Alexa Fluor Plus 488 (Invitrogen, A32731, lot:RJ243417)

Validation: According to manufactures, rabbit polyclonal anti-c-Fos, goat polyclonal anti-GFP, Rabbit polyclonal anti-GFP and Mouse monoclonal anti-TH was validated in mouse for immunofluorescence. These antibodies have been utilized in previous publications to detect rabbit polyclonal anti-c-Fos (Koga et al 2020), goat polyclonal anti-GFP (Sano et al 2018), rabbit polyclonal anti-GFP (Lee et al 2020), chicken polyclonal anti-mCherry (Sagoshi et al, 2020), goat polyclonal anti-Tph2 (Farrelly et al 2019), and mouse monoclonal anti-TH (Jiao et al 2020), and sheep anti-Dig (Choi et al, 2014) in the mouse brain. Alexa488, Alexa594, Alexa680 are generally used secondary antibodies. Concentration of each antibody was determined in our laboratory by analyzing a series of dilutions.

Animals and other organisms

Policy information about studies involving animals; ARRIVE guidelines recommended for reporting animal research

Laboratory animals	Test animals (residents):<br>Mouse, ICR (CD-1) (Charles River Labs), male, 10-15 weeks at the time of behavioral experiments.<br>Mouse, F1 offspring from Tph2-tTA::TetO-eArchT transgenic males (Established by A. Yamanaka and K.F. Tanaka) and CD-1 females, male, 11-16 weeks at the time of behavioral experiments.<br>Stimulus animals (intruders):<br>Mouse, ICR (Japan CLEA), male, more than 10 weeks old. Olfactory bulbectomized at least 1 week before the experiment to eliminate the expression of aggressive behavior from the intruder.<br>Mouse, BALB/cByJ (The Jackson Laboratory), male, more than 10 weeks old.<br>Mouse, C57BL/6J (Japan CLEA), female, 10-12 weeks old.<br><br>Resident males were housed individually in standard mouse cages with corn cob bedding material, and intruder animals were group housed (3 to 5 males per cage) in the same standard mouse cages with corn cob bedding material throughout the experiment. All animals were maintained in the animal rooms with controlled temperature (23±2oC, average humidity; 50%) on a 12 h light/dark cycle with ad libitum access to food and water.
Wild animals	This study did not involve wild animals
Field-collected samples	This study did not involve samples collected from the field
Ethics oversight	Experimental procedures were performed in accordance with the National Institutes of Health Guide for Care and Use of Laboratory animals, the Animal Care and Use Committee at the University of Tsukuba (approval number 20-341, 20-342, 21-304, 21-422), and the Icahn School of Medicine at Mount Sinai (ISMMS) Animal Care and Use Committee (approval number LA10-00266).

Note that full information on the approval of the study protocol must also be provided in the manuscript.
